# Distribution of *Streptococcus pneumoniae* Serotypes in Nasopharyngeal Carriage Among Children in Indonesia and Estimated Coverage of Pneumococcal Conjugate Vaccines: A Systematic Review

**DOI:** 10.3390/vaccines14050451

**Published:** 2026-05-19

**Authors:** Ari Prayitno, Mulya Rahma Karyanti, Nina Dwi Putri, Pratama Wicaksana, Felicia Felicia, Shafira Ninditya, Sarah Kemalasari, Aldila Ardine, Hindra Irawan Satari, Sri Rezeki Hadinegoro

**Affiliations:** 1 Department of Pediatrics, Faculty of Medicine, Dr. Cipto Mangunkusumo National General Hospital, Universitas Indonesia, Jl. Pangeran Diponegoro No. 71, Central Jakarta 10430, Indonesia; karyanti@doctor.com (M.R.K.); ninadwip@gmail.com (N.D.P.); pratamawicaksana@yahoo.com (P.W.); felicia13@ui.ac.id (F.F.); drshafiraninditya@gmail.com (S.N.); hsatari@ikafkui.net (H.I.S.); shadinegoro46@gmail.com (S.R.H.); 2 Medikids Clinic, Jl. Kalimalang/Per Kav PTB Block A2 No. 04, East Jakarta 13450, Indonesia; sarahkemalasari@gmail.com; 3 Jakarta Hospital, Jl. Jend. Sudirman No. Kav. 49, South Jakarta 12930, Indonesia; ardinealdila@gmail.com

**Keywords:** invasive pneumococcal disease, nasopharyngeal carriage, pneumococcal conjugate vaccine, serotype distribution, *Streptococcus pneumoniae*

## Abstract

**Background**: *Streptococcus pneumoniae* may asymptomatically colonize the human nasopharynx and remains a leading cause of invasive and noninvasive disease in children, accounting for an estimated 294,000 global deaths in those aged under five years. Nationally representative serotype data from Indonesia remain limited despite national PCV13 rollout in 2022. This study aims to evaluate the distribution of *Streptococcus pneumoniae* serotypes and estimate the coverage of pneumococcal conjugate vaccines (PCVs) among children aged 0–18 years in Indonesia. **Methods**: Systematic search of PubMed, Scopus, ScienceDirect, Google Scholar, and Paediatrica Indonesiana (to December 2025) for observational studies (PROSPERO CRD420251239935). The extracted data included the study period, setting, population, specimen type, serotypes, sample size, and nasopharyngeal carriage. Pooled serotype prevalence is calculated; vaccine coverage estimated for pneumococcal conjugate vaccines containing 10 (PCV10), 13 (PCV13), 15 (PCV15), and 20 (PCV20) serotypes assuming vaccine-type priority in multicolonization. Risk of bias assessed using the Joanna Briggs Institute’s checklist for prevalence studies. **Results**: Nineteen studies across 13 regions of Indonesia involving children aged 0–18 years included. Nasopharyngeal carriage ranged from 21.0% to 87.6% in healthy children and 9.2% to 73% in children with illnesses. The most common serotypes were 19F, 23F, 6B, 14, 19A, and 34. Non-typeable isolates accounted for more than 20% of all isolates in several studies. The pooled coverage for PCV10, PCV13, PCV15, and PCV20 was 40.3%, 50.2%, 50.8%, and 57.0% respectively. Low-moderate RoB (63% low). **Conclusions**: The dominant serotypes largely included in PCV13. Active surveillance is required to monitor serotype shifts and ensure the long-term effectiveness of the national vaccination program.

## 1. Introduction

*Streptococcus pneumoniae* (pneumococcus) is a Gram-positive coccus that colonizes the human nasopharynx, which may lead to noninvasive (non-IPD) and invasive (IPD) pneumococcal disease, with up to 11% mortality in children under 5 years [1,2]. Nasopharyngeal carriage (NPC) of pneumococcus occurs in healthy children without symptoms, but an impaired immune system enables spread to the lower respiratory tract, adjacent organs, or bloodstream invasion [3]. Risk factors include host immunity, household crowding, recurrent viral infections, and cigarette smoke exposure [4].

Non-IPDs develop when pneumococcus spreads to non-sterile sites, such as the middle ear (otitis media), sinuses (sinusitis), and bronchi (bronchitis). In contrast, IPDs develop when pneumococcus invades normally sterile sites, including the blood, cerebrospinal fluid (CSF), pleura, joints, or pericardium. IPD is associated with high morbidity and mortality in children and immunocompromised adults [5].

In 2015, pneumococcus was estimated to cause approximately 294,000 deaths among children aged under five years worldwide, with about 87,200 of these deaths occurring in the Southeast Asia region [6]. IPDs occur in 40% of children aged under two years, with an estimated incidence of 6.4 cases per 100,000 population in Europe in 2018 [7]. A study in Canada between 1999 and 2019 reported that 76.6% of IPD cases occurred in children aged under 5 years, with bacteremia accounting for 59.2% of IPD cases and meningitis accounting for 8.8% [8]. In contrast, a study in China reported that bacteremia accounted for 37.7% of IPD cases and meningitis accounted for 32.4% [9]. The composition of the polysaccharide capsule surrounding the pneumococcus creates different serotypes, with 100 currently known [10]. As of 2018, 6–11 pneumococcal serotypes, including 6A, 6B, 14, 19A, 19F, 1, 7F, and 23F, have been identified as the primary causes of IPD in children. The spectrum of pneumococcal serotypes responsible for IPDs continues to expand over time [11,12].

The most effective strategy for preventing IPDs is vaccination. Currently, there are two types of vaccines: the pneumococcal polysaccharide vaccine (PPSV) and the pneumococcal conjugate vaccine (PCV). The PPSV is not administered to children aged under 2 years because it is non-immunogenic; instead, they receive the PCV, as it is conjugated to an immunogenic protein, elicits a memory cell response, and provides long-term protection [13]. PCV formulations have evolved progressively: PCV7 (2000) contained seven serotypes (4, 6B, 9V, 14, 18C, 19F, 23F); PCV10 (2009) added 1, 5, 7F; PCV13 (2010) added 3, 6A, 19A; PCV15 (2021) added 22F, 33F; and PCV20 (2022) added 8, 10A, 11A, 12F, 15B [14,15,16,17,18].

In Indonesia, PCV13 was introduced through a demonstration program in 2017, expanding nationally and incorporated into Indonesia’s routine immunization schedule since September 2022, with a 2p + 1 regimen (primary doses at two and three months of age, followed by a booster at 12 months) [19]. Multiple studies report regional serotype variation: Lombok 1997 found 48.0% NPC with serogroups 6, 23 dominant [20]; Bali study showed 19F predominance (66.7%), followed by 23F (9.5%), 6A/B (9.5%), 7F (4.8%), 15B/C (4.8%); 38.1% non-typeable [21]; Prof. Ngoerah General Hospital (Bali) also found 19F (32%) dominant, then 3/6A/B (9%), 33F/15B/C (3%) [22]. Serotypes 6A/B, 23F, and 19F are the predominant strains identified in the ten member countries of the Association of Southeast Asian Nations (ASEAN), whereas 6A/B, 23F, 19F, 19A, and 3 were the predominant serotypes in Indonesia [23,24]. However, pre- and early post-introduction serotype data remain fragmented.

This systematic review synthesizes Indonesian pediatric serotype data published through December 2025 by health status (healthy/ill), specimen type (carriage), and geography to estimate PCV10/13/15/20 coverage and identify surveillance gaps (PROSPERO CRD420251239935). Serotype replacement post-PCV introduction is anticipated; baseline data are essential for optimal formulation selection and monitoring in this high-burden setting.

## 2. Materials and Methods

This systematic review was conducted and reported in accordance with the Preferred Reporting Items for Systematic Reviews and Meta-Analyses (PRISMA) guidelines. The study protocol was registered on PROSPERO (CRD420251239935). PubMed, Scopus, ScienceDirect, Google Scholar, and Paediatrica Indonesiana were systematically searched on 9 December 2025 using a combination of search terms joined using Boolean operators: (*Streptococcus pneumoniae* OR pneumococcus OR pneumococcal OR pneumoniae) AND (serotype OR serogroup) AND (Indonesia), which were then adjusted to each database.

The identified articles were screened based on their titles, abstracts, and the availability of their full texts for review. The inclusion criteria were: (1) articles published in either Indonesian or English, (2) full-text available, (3) published up to 2025, (4) studies conducted in Indonesia, (5) studies involving children aged 0–18 years, and (6) availability of data presenting the pneumococcal serogroup or serotype distribution. The exclusion criteria were: (1) studies involving non-human subjects or in vitro experiments, (2) systematic reviews and meta-analyses, and (3) duplicate publications. No language restrictions applied beyond full-text availability.

**Figure 1:**
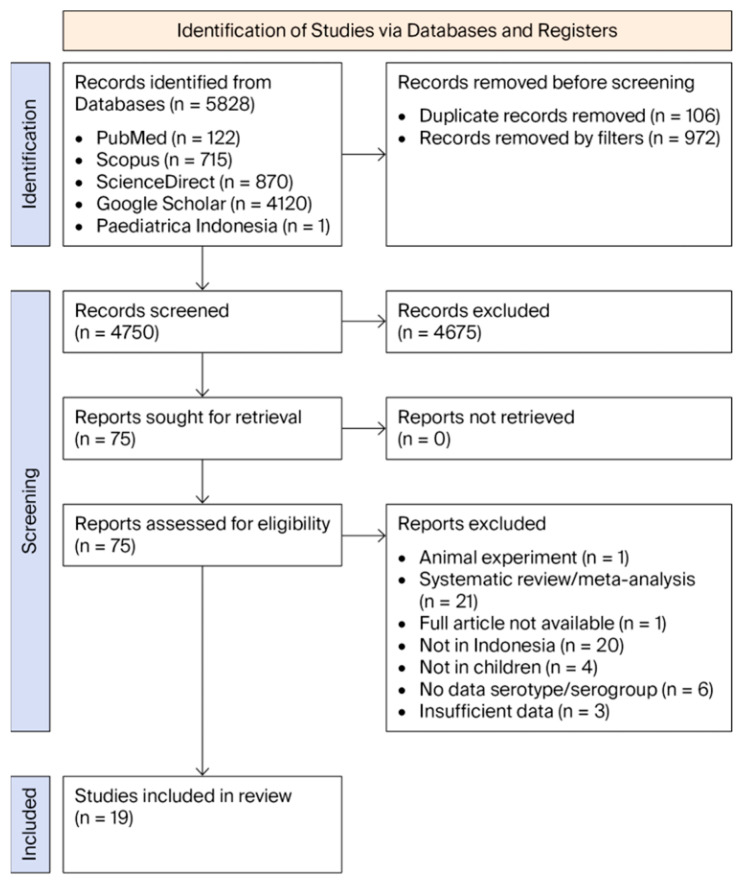
Flow diagram of study identification and screening, with 19 original studies included.

Following the PRISMA guidelines, studies were selected based on title, abstract, and full-text review according to predefined inclusion criteria (Figure 1). Discrepancies were resolved by consensus or a fifth adjudicator (AP). Methodological quality was assessed by four independent reviewers (AA, F, SN, SK) using the Joanna Briggs Institute (JBI) Checklist for Prevalence Studies. The detailed item-level results of the JBI checklist for each included study are presented in Table S1. Risk of bias at the study level was assessed using the JBI checklist, while the overall certainty of evidence was evaluated separately using a Grading of Recommendations Assessment, Development and Evaluation (GRADE)-adapted framework. Studies were categorized as low (≥7 “Yes”), moderate (4–6), or high risk of bias (≤3). The certainty of the body of evidence for each primary outcome (pooled serotype prevalence and estimated vaccine coverage) was assessed using the GRADE-adapted approach for prevalence and coverage evidence, rather than the standard intervention-effect GRADE framework. This adapted approach was applied because the outcomes represent descriptive prevalence and vaccine coverage rather than intervention effects.

Extracted data included study period, setting, population, specimen type, and serotype distribution. Serotypes were classified as vaccine-type (VT), non-vaccine-type (non-VT), or non-typeable (NT) according to their inclusion in PCV10, PCV13, PCV15, and PCV20 formulations. Estimated PCV serotype coverage was defined as the proportion of pneumococcal isolates or serotype-positive detections whose serotypes were included in each PCV formulation. The denominator was the total number of pneumococcal isolates or detections with available serotype information, and the numerator was the number classified as vaccine-type for PCV10, PCV13, PCV15, or PCV20.

Multicolonization was defined as the detection of two or more pneumococcal serotypes within a single sample. In studies reporting multiple serotypes per individual, a vaccine-type (VT) priority approach was applied for vaccine coverage estimation. Specifically, if at least one detected serotype was included in a given PCV formulation, the isolate was classified as vaccine-type for that formulation; otherwise, it was classified as non-vaccine-type. This approach was applied to minimize potential underestimation of vaccine coverage in the presence of multicolonization.

The primary pooled analysis was restricted to nasopharyngeal carriage (NPC) studies. Studies based exclusively on blood or cerebrospinal fluid isolates were retained in the systematic review but excluded from the primary pooled NPC serotype coverage analysis and summarized descriptively, because they represented clinically distinct invasive pneumococcal disease populations and included very few pneumococcal isolates. For studies including both children and adults, only child-specific data were used when available. Descriptive pooled proportions were calculated by summing vaccine-type isolates or detections across eligible NPC studies and dividing by the summed denominator. Ninety-five percent confidence intervals were calculated using the Wilson score method. Subgroup estimates were calculated separately for healthy children and children with illnesses.

Forest plots were generated for PCV13 and PCV20 to visualize study-level estimates. All eligible NPC study groups (*n* = 17) provided sufficient data to calculate study-level proportions and corresponding confidence intervals were therefore included in the forest plot analysis.

As a sensitivity analysis, study-level proportions were logit-transformed and synthesized using a random-effects inverse-variance model. Model-based pooled estimates and 95% confidence intervals were back-transformed to proportions for interpretation. Between-study heterogeneity was assessed using Tau^2^, Chi^2^, and I^2^ statistics. Descriptive pooled proportions and Wilson confidence intervals were calculated using Microsoft Excel. Random-effects sensitivity analyses were conducted in Review Manager version 5.4 using generic inverse variance models.

Pneumococcal multicolonisation was frequently reported, with vaccine serotypes prioritized in mixed classifications. In such cases, VTs are of primary concern. Three possible patterns of pneumococcal multicolonisation were identified: (1) VT with VT in a single specimen, classified as VT; (2) non-VT with non-VT in a single specimen, classified as non-VT; and (3) VT with non-VT in a single specimen, classified as VT. For serotypes reported with a ‘/’ symbol, classification into VT or non-VT was determined based on the unresolved serotypes: (1) if all undifferentiated serotypes were VTs, they were classified as VT (e.g., 6A/6B was considered VT, whereas 35A/35C/42 was considered non-VT), and (2) if at least one of the unresolved serotypes was a VT, the entire group was classified as VT (e.g., 9V/9A, where 9V is a VT and 9A a non-VT, was considered VT).

## 3. Results

The searches identified 5828 articles: 122 in PubMed, 715 in Scopus, 870 in ScienceDirect, 4120 in Google Scholar, and one in Paediatrica Indonesiana (Figure 1). After screening these articles against the inclusion and exclusion criteria, 19 original research articles were eligible for inclusion: Nine studies involved children with illnesses, nine healthy children, and one both populations.

A total of 17 NPC study groups contributed to the primary serotype coverage analysis. Some studies reported results separately for different populations (e.g., healthy and children with illnesses) and were therefore treated as independent in study group analyses. Of the 17 NPC study groups, all were included in the forest plot analysis for PCV13 and PCV20, as sufficient data were available for study-level estimates. Three blood or CSF-based IPD studies were retained for descriptive synthesis only because they represented clinically distinct invasive disease contexts and included very few pneumococcal isolates. In studies enrolling both children and adults, only child-specific data were used when available.

The 19 included studies (Table 1) were conducted across 13 regions of Indonesia, and most studies were conducted in Western Indonesia, while no studies were identified from Eastern Indonesia.

The prevalence of nasopharyngeal carriage (NPC) among healthy children ranged from 21.0% to 87.6%, with the highest prevalence reported among children under 5 years of age [24]. Among children with illnesses, NPC prevalence ranged from 9.2% to 73.0%, with higher prevalence observed in conditions such as acute otitis media, human immunodeficiency virus (HIV) infection, and acute respiratory tract infections (ARTIs), while lower prevalence was reported in children with pneumonia.

**Figure 2:**
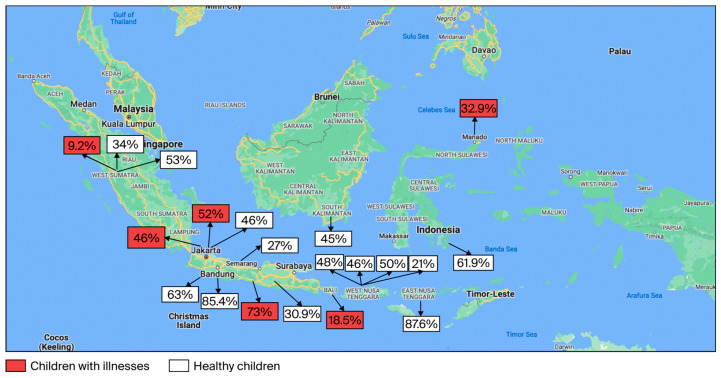
Distribution map of the prevalence of pneumococcal NPC in Indonesia [20,24,25,26,27,28,29,30,31,32,33,34,35,36,37,38,39,40,41].

Most studies were conducted in Western Indonesia, with limited data from the Central regions, and no studies were identified from Eastern Indonesia. This uneven geographic distribution may limit the representativeness of national estimates. The geographic distribution of NPC prevalence is illustrated in Figure 2, while detailed study characteristics are presented in Table 1.

Pneumococcal serotype distribution demonstrated a predominance of VTs, particularly 19F, 23F, 6B, and 14. Nearly all studies reported the presence of serogroups 6, 19, and 23. Additional VTs identified were 19A (12 studies), 3 (11 studies), and 6A (seven studies) in PCV13; 22F (three studies) and 33F (one study) in PCV15; and 11A (eight studies), 10A (four studies), 12F (four studies), 8 (two studies), and 15B (one study) in PCV20. Serotype 34 was also frequently reported and should be noted as a non-vaccine serotype not included in PCV10, PCV13, PCV15, or PCV20. Non-typeable (NT) isolates were common in several studies, as documented by Hadinegoro et al. [28] (21.0% of all non-VTs), Salsabila et al. [34] (14.0%), and Safari et al. [27] (26.0%).

The overall descriptive pooled coverage was 40.2% (95% CI: 38.4–42.1) for PCV10, 50.2% (95% CI: 48.3–52.0) for PCV13, 50.8% (95% CI: 49.0–52.7) for PCV15, and 57.0% (95% CI: 55.2–58.9) for PCV20 (Table 2). Coverage estimates were lower among children with illnesses than among healthy children, although this comparison should be interpreted cautiously because the illness subgroup included fewer isolates/detections and clinically heterogeneous populations. The dominant serotypes in children with illnesses were 19F, 6B, and 23F which are included in PCV13. Notably, a very limited number of invasive pneumococcal disease (IPD) cases were identified. Among these, serotypes 6B and 7F were reported; however, these findings should be interpreted with caution due to the extremely small number of isolates.

Several studies had limitations in accurately determining pneumococcal serotypes. Some serotypes are difficult to distinguish, such as 6A/6B or 15B/15C, due to constraints in the laboratory techniques employed. Some studies reported combined 6A/6B serotype [26,27,28,32,34,35,36], whereas others differentiated them individually. These differences in serotype resolution contributed to heterogeneity across studies and affected the precision of estimated PCV serotype coverage.

Pneumococcal multicolonization was reported in several included studies and is summarized in Table 3, including detection methods and study characteristics [20,24,27,28,35,37,39].

**Figure 3:**
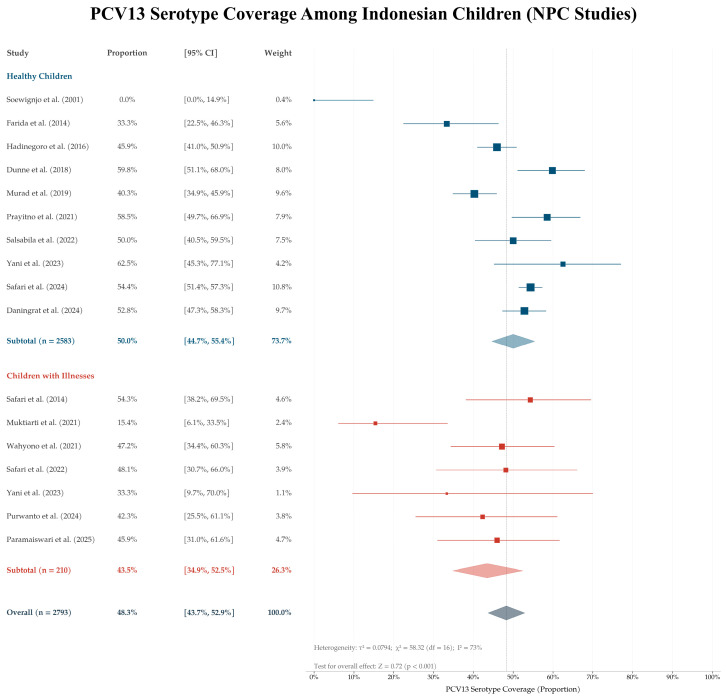
Study-level estimated PCV13 serotype coverage among 17 nasopharyngeal carriage study groups. Blue indicates healthy children, while red indicates children with illnesses [20,24,25,26,27,28,29,30,31,32,33,34,35,36,37,38,39,40,41].

Random-effects sensitivity analyses were conducted for PCV13 and PCV20 using logit-transformed study-level proportions. The PCV13 sensitivity analysis showed substantial variability across NPC studies (Figure 3). The back-transformed pooled estimate was 48.3% (95% CI: 43.7–52.9), with substantial between-study heterogeneity (I^2^ = 73%). This estimate was broadly consistent with the descriptive pooled estimate in Table 2, although the confidence interval was wider.

**Figure 4:**
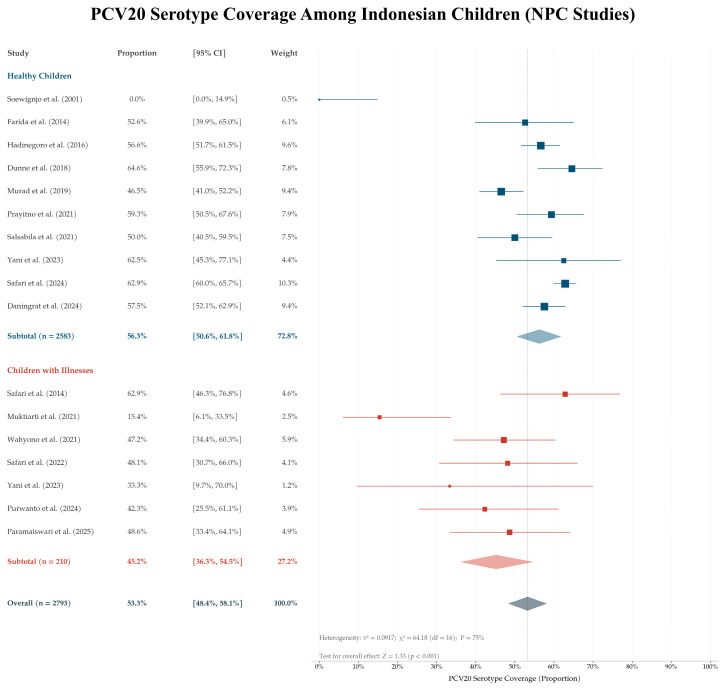
Study-level estimated PCV20 serotype coverage among 17 nasopharyngeal carriage study groups. Blue indicates healthy children, while red indicates children with illnesses [20,24,25,26,27,28,29,30,31,32,33,34,35,36,37,38,39,40,41].

The PCV20 sensitivity analysis also showed substantial variability across NPC studies (Figure 4). The back-transformed pooled estimate was 53.3% (95% CI: 48.4–58.1), with substantial between-study heterogeneity (I^2^ = 75%). This estimate was lower than the descriptive pooled estimate in Table 2, but the overall pattern remained consistent, showing higher estimated serotype coverage with a higher-valency PCV formulation. The substantial heterogeneity observed in both sensitivity analyses supported the decision to retain descriptive pooled proportions as the primary summary estimates.

Risk of bias assessment using the Joanna Briggs Institute (JBI) Checklist showed that 12 studies (63.2%) had low risk, 6 (31.6%) had moderate risk, and 1 (5.2%) had high risk of bias (Table 4). Detailed item-level assessments are provided in Table S1. The JBI checklist evaluates methodological quality at the individual study level, whereas the GRADE-adapted assessment reflects the overall certainty of the body of evidence. The overall certainty of evidence for serotype distribution and PCV coverage was assessed using a GRADE-adapted approach, as described in the Methods, and was rated as LOW due to observational study design, moderate risk of bias (including one high-risk study), and substantial to considerable heterogeneity (I^2^ = 75–78%). NPC prevalence ranged from 21.0% to 87.6% across studies, reflecting substantial between-study variability. Two reviewers judged independently. This is particularly relevant in settings such as Indonesia, where data on pneumococcal serotype distribution remain limited.

## 4. Discussion

### 4.1. Prevalence of Pneumococcal NPC in Healthy Children

Based on the included studies, the prevalence of pneumococcal NPC among healthy children in Indonesia ranged from 21.0% to 87.6%, with a mean of 48.8%. This prevalence is higher than that reported by a systematic review and meta-analysis of studies conducted between 2001 and 2019 in Southeast Asia (36%, 95% CI: 32.4–37.8%) [2]. Several factors may contribute to the higher prevalence in Indonesia, including its geographical status as an archipelagic country with diverse climates and rainfall patterns influencing humidity levels, as well as demographic characteristics such as age distribution and socioeconomic status [42,43].

### 4.2. Association Between Demographic Characteristics and Pneumococcal NPC

Regarding age, the prevalence of pneumococcal NPC in children decreases with age. This finding is consistent with Wang et al. [5], who examined 1702 preschool children in Liuzhou, China, reporting that children aged 5–7 years had a lower risk of pneumococcal NPC compared to those aged 2–<5 years (odds ratio [OR]: 0.55, 95% CI: 0.40–0.76, *p* = 0.001). In a systematic review and meta-analysis of studies conducted in Southeast Asia, Daningrat et al. [2] identified age as a significant risk factor for pneumococcal NPC in three studies. Age was identified as a risk factor in studies conducted in Lombok (*p* = 0.041) and Semarang, where children aged 6–60 months were at greater risk of pneumococcal NPC compared to adults aged 45–70 years (OR: 7.7, 95% CI: 1.5–13.0) [20,26,28]. Several studies have shown that the prevalence of pneumococcal NPC is highest in children aged under five years.

Dunne et al. [44] reported that infants aged 5–8 weeks who lived in households with at least two children aged under five years and a low income were at increased risk of pneumococcal NPC. Among children aged 12–23 months, acute respiratory infection symptoms, cohabitation with at least two children aged under five years, and residing in rural areas were associated with an increased risk of pneumococcal NPC. According to Fadlyana et al. [45], additional risk factors for pneumococcal NPC included living with at least two children aged under 5 years and having symptoms of upper respiratory tract infection. Other factors included acute respiratory infection, ongoing breastfeeding, residing in rural areas, and cesarean delivery. The prevalence of pneumococcal NPC was higher among children with (76.0%) than without (60.0%) exposure to cigarette smoke [46].

### 4.3. Interpretation of Subgroup Coverage

The pooled vaccine coverage for PCV13—the formulation currently utilized in Indonesia’s national immunization program—was notably lower among children with illnesses (43.7%) compared to their healthy counterparts (50.7%). This disparity persisted across expanded formulations, with PCV20 coverage reaching 57.9% in healthy children but only 45.5% in the illness group. The reduced coverage in the illness subgroup suggests that non-vaccine serotypes (NVTs) play a significant role in the pediatric disease burden. For instance, in children with acute otitis media (AOM), serotype 23A was frequently identified, contributing to a high nasopharyngeal carriage rate of 73.0% that falls outside the protection of PCV13 [35]. Additionally, the high prevalence of non-typeable (NT) isolates—exceeding 20% in several studies—indicates a diverse pneumococcal landscape that current vaccines and standard PCR methods may not fully address.

These findings highlight a “protection gap” for the most vulnerable children. While the national rollout of PCV13 is a vital step, the dominance of certain NVTs and the potential for serotype replacement necessitate vigilant, long-term surveillance. Moving forward, these data suggest that transitioning to higher-valent vaccines, such as PCV20, may be essential to bridge this gap and ensure the sustained success of Indonesia’s immunization strategy.

### 4.4. Dominant Serotypes and Increased Risk of Pneumococcal NPC

The most frequently identified pneumococcal serotypes in the included studies were 19F, 23F, 6B, 14, 19A, and 34. Among these, serotypes 19F, 23F, 6B, 14, and 19A are classified as VT, whereas serotype 34 is categorized as NVT. A similar pattern of predominant VT serotypes (19F, 23F, 14, and 6B) has been reported in a study conducted in Malaysia [47]. This similarity may be attributable to comparable study populations—primarily healthy children aged 0–5 years—as well as shared risk factors observed in both countries, such as exposure to cigarette smoke and a history of upper respiratory tract infection or otitis media [48]. For instance, in children with acute otitis media (AOM), serotype 23A was frequently identified. The overall nasopharyngeal carriage (NPC) rate was 73.0%, while PCV13 serotypes accounted for 41% of the isolates [35]. The dominant serotypes identified in the included studies also resembled those found in Thailand, including 6B, 14, 18C, 19F, and 23F [49]. A common risk factor reported in studies conducted in both Indonesia and Thailand was contact with other children aged under 5 years [49]. The study conducted in Semarang, Indonesia, reported an increased risk of pneumococcal NPC in households with young children (OR: 3.0, 95% CI: 1.9–4.7) [26]. Similarly, a study conducted in Thailand indicated that cohabitation with other children was associated with pneumococcal NPC (OR: 1.4, 95% CI: 1.0–1.9) [49]. Additionally, a multicentre study conducted in Vietnam, Singapore, Thailand, Malaysia, and the Philippines reported that daycare attendance increased the risk of pneumococcal NPC (OR: 1.6, 95% CI: 1.2–2.2, *p* = 0.003) [2].

Importantly, the identification of serotype 34 as an NVT highlights the presence of serotypes not covered by current pneumococcal conjugate vaccines, including PCV10, PCV13, PCV15, and PCV20. This underscores the potential for serotype replacement and strongly emphasizes the critical importance of continuous, high-quality, and region-specific surveillance systems to monitor changes in serotype distribution over time. Such surveillance is essential to detect emerging non-vaccine serotypes early, evaluate the real-world impact of vaccination programs, and provide timely evidence to guide updates in vaccine policy and formulation. In contrast, a multinational study conducted in nine European countries found different patterns, with pneumococcal serotype 11A most frequently identified, followed by 23A and 19A [1]. These discrepancies suggest that global patterns may not accurately predict vaccine impact in Indonesia. Ultimately, they highlight the necessity of using region-specific data to ensure the national immunization strategy is effectively calibrated to the local burden.

The dominant pneumococcal serotypes identified in Indonesia, including 19F, 23F, 6B, 14, and 19A, have also been reported in pneumococcal carriage and invasive pneumococcal disease studies from other settings, although their distribution varies by geography, population, and vaccination context [50,51]. These five serotypes have been consistently reported across multiple studies in Indonesia [27,34,36,42]. Although the available studies span more than two decades, from Soewignjo et al. [20] to Rani et al. [41], temporal trends should be interpreted cautiously because of differences in study methods, locations, populations, and study periods. The continued detection of these serotypes may partly reflect limited PCV use before national PCV introduction. However, the current evidence is insufficient to confirm a stable national serotype pattern over time. Consequently, coverage remains low, leaving many individuals unprotected. As a result, pneumococcal NPC patterns have not significantly shifted, since overall vaccine coverage, especially for the full three-dose schedule, remains insufficient to establish herd protection, which would otherwise alter colonization dynamics in the population. The studies included in this systematic review identified pneumococcal serotypes included in PCV20, with serotype 11A documented in 6/10 (87.5%) studies involving healthy children [24,26,28,29,30,39]. Regarding the pneumococcal serotypes included in PCV15, one study reported 22F and 33F [28] and while two other studies reported 22F the same serotype [24,30].

### 4.5. Geographic Distribution and Dynamics of Pneumococcal Serotypes

Epidemiological research on pneumococcus in Indonesia began in 2001 with a study conducted in Lombok [20]. Between 2012 and 2019, research expanded to Western Indonesia, including Jakarta, Semarang, Bandung, and Padang. From 2020 to the present, research has further expanded to other regions, including Central Indonesia (Kotabaru, Tabanan, Southwest Sumba, Manado, and Wakatobi) and additional western regions (Banyumas, Gunungkidul, and Surabaya).

Three of the included studies had been conducted in Lombok [20,28,32]. The first, conducted in 2001, sixteen serogroups were identified that could not be further differentiated as serotypes. The subsequent study, conducted in 2016, identified 10 serotypes, all of which were included in PCV13 (6A/6B, 19F, 23F, 19A, 14, 3, 4, 7F, 1, and 9V). The most recent study, conducted in 2021, identified eight serotypes, all of which were again included in PCV13 (6A/6B, 19F, 23F, 14, 3, 19A, 1, and 5). Of the four dominant serotypes in Indonesia, three (6A/6B, 19F, and 23F) were consistently detected in Lombok, all of which are included in PCV10, PCV13, and PCV15. Before 2014, the ability to identify pneumococcal serotypes was limited, allowing only serogroup identification (e.g., serogroups 6, 19, and 23). However, since 2018, studies have identified serotypes with greater specificity; for instance, the study conducted in Bandung distinguished 6A from 6B [29]. The most recent study conducted in 2024 in Southwest Sumba and Gunungkidul was able to differentiate pneumococcal serotypes with even greater detail [24].

The prevalence of pneumococcal NPC among healthy children in Indonesia varies across regions, with no clear increasing or decreasing trend from west to east, or vice versa. This variability may be attributed to the limited availability of research data, which is concentrated mainly in the central and western regions and often restricted to major cities, likely due to resource availability and the presence of local laboratory facilities capable of analyzing pneumococcal NPC and serotypes. Additionally, the determination of pneumococcal serotypes has become increasingly refined in Indonesia over the years, driven by advances in diagnostic techniques. Serotype identification initially relied on the Quellung reaction, which requires specialized expertise and technical skills, and was later supplemented by polymerase chain reaction (PCR)-based methods. However, the introduction of whole genome sequencing (WGS) has enabled not only serotype identification but also the genotypic characterization of pneumococcal isolates [52,53]. In Indonesia, serotyping is currently limited to the Quellung and PCR-based methods. Consequently, some isolates remain NT due to methodological limitations [24]. However, the use of more advanced techniques could enable the serotype of these NT isolates to be identified. Future studies should prioritize the characterization of these currently unclassified strains to more accurately map Indonesia’s pneumococcal landscape and monitor for potential vaccine escape.

### 4.6. Distribution of Pneumococcal Serotypes in Children with Illnesses

Globally, an estimated 8,910,000 cases of pneumococcal pneumonia occurred in 2015, including approximately 4,280,000 cases in Southeast Asia. IPDs account for 44.0% of mortality globally and 39.0% of mortality in Southeast Asia [6]. In Indonesia, studies have shown that in children with illnesses, the most common VTs are 19F, 23F, 14, 3, and 19A, and the most common non-VTs are 23A, 35B, 34, 6C, and 16F [25,27,31,33,35,36,37,38,40,41]. Differences in serotype distribution are also observed in neighboring countries, such as Malaysia, where the most frequent VTs are 19F, 23F, 14, 6B, and 19A, and the most frequent non-VTs are 23A, 6C, and 13 [47,48]. At the regional level in Southeast Asia, the most common serotypes are 1, 3, and 4 in IPD cases and 3, 34, and 1 in non-IPD cases [54]. This contrasts with those found in Europe, where the dominant serotypes in IPD cases are 8, 3, and 19A [7]. Such differences may be attributable to factors such as climate, temperature, air quality, antibiotic use, as well as the coverage and type of PCVs implemented in each country. In addition, Europe’s third-dose (PCV3) coverage across the region reached 86% by 2024 (data from WHO), with many countries exceeding 90%. This high uptake has effectively suppressed traditional vaccine-type IPD, though the persistence of serotypes 3 and 8 has recently driven many European nations to transition toward higher-valency formulations, such as PCV15 and PCV20 [7].

Studies investigating the distribution of pneumococcal serotypes by disease severity among Indonesian children remain limited. Nevertheless, the presence of specific serotypes in carriages often serves as a precursor to severe clinical manifestations. Serotype 3, in particular, is consistently associated with severe clinical manifestations of pneumococcal disease, including empyema, bacteremia, cardiotoxicity, and meningitis, with mortality rates ranging from 30% to 47% [55]. The NPC rate of serotype 3 is estimated at 30–70% among healthy individuals [56]. The NPC rate of serotype 3 (30–70%) reported in [56] is derived from a human challenge study and may not reflect population-based carriage estimates. Consequently, serotype 3 warrants close monitoring despite the limited data available from Indonesia. NPC studies in healthy Indonesian children have reported serotype 3 as one of the dominant serotypes, including those in Gunungkidul and Banyumas [24,35]. However, because these findings are derived from studies of healthy children, the specific contribution of serotype 3 to the clinical burden of IPD in Indonesia remains inconclusive and requires validation through hospital-based surveillance. Sari et al. [57] concluded that serotype 3 has a genomic profile that may enhance its ability to evade PCV-induced immunity, as one isolate belonging to lineage *SPN3 ST700-GPSC10* demonstrated vaccine escape potential. However, this lineage was identified in only a single isolate, highlighting the need for further research. This high carriage prevalence in the community represents a significant reservoir for potential severe pediatric disease.

Studies investigating the distribution of pneumococcal serotypes by disease severity among Indonesian children remain limited. Nonetheless, some clinical data provide preliminary insights. The prevalence of pneumococcal NPC among children with acute otitis media in Indonesia was estimated at 73.0%, higher than the prevalence reported in Thailand (46.0%) and Japan (31.7%) [17,35,49]. The most common serotypes among Indonesian children with otitis media were 23A, 6A/6B, and 3, compared to 19F, 14, and 3 in Thailand, and 19F, 6B, and 23F in Japan [17,35,49]. Among children with chronic conditions such as HIV infections, the prevalence of pneumococcal NPC in Indonesia was 46–52%, with dominant serotypes 19F, 23F, and 6A/6B, substantially higher than in Istanbul (10.4%), where the dominant serotypes were 6A/B/C, 19F, and 15B/C [11,33]. For more severe conditions, such as meningitis, the prevalence of pneumococcal NPC among Indonesian children with IPDs was 2%, higher than among Cambodian children with IPDs (0.4%). The pneumococcal serotypes identified were 6B and 7F among Indonesian children with meningitis and 1, 6B, and 19A among Cambodian children with meningitis [25,31,58]. These findings suggest that NPC by specific pneumococcal serotypes plays an essential role in the pathogenesis of pneumococcal disease, ranging from mild conditions such as otitis media to severe conditions such as meningitis.

Regarding IPDs, the pneumococcal serotype 7F was identified in Indonesian children with meningitis, bilateral lobar pneumonia, and sepsis, and 6B in those with bacterial meningitis [25,31]. These findings differ from the serotype patterns observed in neighboring countries. Most IPD cases are caused by serotypes 6B, 23F, 14, 19A, and 19F in Thailand and Malaysia, and by serotypes 6B, 19F, 14, 1, 18C, and 23F in the Philippines [47,49,54]. The differences observed between countries can largely be attributed to the limited availability of research data in Indonesia, which currently comprises only two studies on pneumococcal serotypes associated with pediatric IPD cases in Jakarta. These two studies identified only two serotypes (6B and 7F) from CSF and blood specimens, both of which are VTs. However, these findings should be interpreted with extreme caution, as the serotype distribution cannot be reliably inferred from such a very small number of cases. These findings highlight the limited nature of pneumococcal serotype data in Indonesian children with IPDs, yet its data cannot be representative to support our conclusion due to its limited data. Such limitations stem from challenges in specimen collection from affected organs, such as CSF, sputum, or blood, which are influenced by the availability of clinical facilities, diagnostic procedures, and parental consent [25,31].

### 4.7. Impact of the National PCV Immunization Program on the Serotype Distribution

The distribution of pneumococcal serotypes in a given country is strongly influenced by national immunization policies and coverage. For instance, the introduction of PCV13 and PCV10 in various countries led to shifts in the dominant serotypes. In Europe, the predominant serotypes included 3, 12F, 19A, and 10A in countries using PCV13, and 19A, 3, and 14 in countries using PCV10 [1]. Colonization by serotypes 10A, 12F, 22F, and 33F increased over time, prompting the development of next-generation PCVs. PCV20 was introduced in Europe and the United States in 2018, aiming to broaden protection against IPDs, including serotype 19A and five additional serotypes whose prevalence increased after the introduction of the PCV [59].

In Indonesia, the distribution of pneumococcal serotypes has shown a tendency toward the dominance of serotypes in PCV13, such as 19F, 19A, and 3. In Indonesia, pneumococcal vaccination was adopted nationally only in 2022, with PCV13 incorporated into the routine childhood immunization program. However, as its implementation is still relatively recent, data regarding the impact of pneumococcal vaccination on changes in national serotype colonization patterns remain unavailable. Although PCV20 received regulatory approval in Indonesia in October 2024, no studies have yet evaluated its effectiveness or the post-introduction shifts in the serotype distribution [60]. Although serotype 3 is covered by PCV13, the vaccine’s effectiveness is lower than that of other serotypes, where vaccine coverage is different than vaccine effectiveness.

Based on a review of several national studies, the proportion of serotypes covered by the available PCVs in Indonesia was estimated at 40.3% for PCV10, 50.2% for PCV13, 50.8% for PCV15, and 57.0% for PCV20. However, evaluation of PCV15 remains limited; three studies conducted in Lombok, Kotabaru, and Banyumas reported the presence of serotype 22F, without the ability to distinguish it specifically from 22A [32,34,35]. Additionally, twelve studies conducted in Semarang, Jakarta, Lombok, Bandung, Padang, Kotabaru, Banyumas, Tabanan and Wakatobi reported the presence of serotype 15B—a component of PCV20—although it could not be differentiated from 15C due to methodological constraints [26,30,32,33,34,35,36,37,39].

Altogether, studies conducted in various regions of Indonesia have identified all serotypes included in PCV20 in the nasopharynx of children. These findings highlight the high potential relevance of PCV20 for immunizing children in Indonesia, and also indicate the need for longitudinal studies to evaluate the direct impact of vaccination on shifts in pneumococcal serotypes over time.

### 4.8. Study Limitations

This systematic review had several limitations that should be taken into account when interpreting its findings. Firstly, limitations prevail in pneumococcal serotyping methods, particularly in differentiating specific serogroups that are difficult to distinguish, including 6A/6B, 15B/15C, 6C/6D, 11A/11D, 35A/35C/42, 18C/18F/18B, 15A/15F, 35F/47F, 7C/7B/40, 22F/22A, 38/25F/25A, 9V/9A, and 33F/33A/37. These limitations are related to variations in laboratory capacity and the diagnostic methods employed in the included studies.

While the Quellung reaction is considered the gold standard for pneumococcal serotyping due to its high accuracy and ability to differentiate all serogroups, this method is costly and requires specialized expertise. In Indonesia, resource and infrastructure constraints restrict the availability of this test to only a few laboratories, such as those located in Jakarta and Bandung.

As an alternative to the Quellung reaction, PCR is a cost-efficient and relatively easy-to-implement method. However, it has limitations in differentiating certain specific serotypes, which may affect the accuracy of results. PCR testing is also typically performed sequentially based on serotype prevalence, potentially leading to fewer common serotypes being overlooked. It tends to detect carriage and multicolonization more, yet culture makes its detection lower. While WGS-based serotyping is now available and offers higher accuracy in identifying both pneumococcal serotypes and genotypes, its implementation remains limited in Indonesia due to its very high cost and infrastructure constraints.

Secondly, heterogeneity in sample characteristics (e.g., various age groups) and laboratory capacity across regions represents another limitation. Laboratories capable of performing Quellung and PCR testing are concentrated mainly in Western Indonesia, particularly in Jakarta and Bandung. In central regions such as Lombok and Kotabaru, no laboratories can currently conduct these tests; therefore, all specimens from these regions must be sent to facilities in Jakarta or Bandung for testing. A notable limitation is the high prevalence of non-typeable (NT) isolates, which exceeded 20% in several studies. These strains, likely representing non-encapsulated *S. pneumoniae* or serotypes beyond the reach of standard PCR primers, were excluded from coverage calculations. Consequently, pooled coverage for PCV10, PCV13, PCV15, and PCV20 may be slightly underestimated. Conversely, the “vaccine-type priority” rule used for unresolved serogroups (e.g., 6A/6B, 15B/15C) may introduce a degree of coverage overestimation. These methodological constraints highlight the urgent need for advanced techniques like whole genome sequencing (WGS) to accurately map Indonesia’s pneumococcal landscape.

Thirdly, challenges in specimen collection represent a further limitation. The skills of healthcare personnel in performing optimal specimen collection varied across regions. High rates of antibiotic use before specimen collection may reduce the likelihood of successful bacterial isolation, thereby increasing the risk of false-negative results. Additionally, physicians’ refusal to perform CSF culture due to clinical considerations and parents’ refusal to consent to invasive procedures pose barriers to specimen collection and testing. Moreover, some studies collected specimens only from children with illnesses, in accordance with their focus on infection; thus, their findings do not yet reflect the serotype distribution in healthy children.

Lastly, an active and continuous pneumococcal surveillance system has not yet been established in Indonesia, e.g., data from the East side of Indonesia, children with IPD, causing a potential evidence gap, limiting the availability of representative and consistent data, both in healthy children and in children with illnesses, which should otherwise serve as the foundation for planning preventive interventions.

Despite these limitations, our systematic review is among the most comprehensive investigations to date on pneumococcal serotype data in Indonesia. To our knowledge, only one systematic review on the pneumococcal serotype distribution in Indonesia has been previously published [2]. Therefore, our findings are expected to serve as an essential reference for policy development, the design of preventive programs, and the planning of more effective pneumococcal vaccination strategies in Indonesia.

### 4.9. Recommendation

We have several recommendations regarding the limitations above. First, active surveillance in Indonesia is needed, especially in the Eastern side of Indonesia, to complete the data. Secondly, an evaluation regarding PCV13 rollout needs to be published (coverage and national statistics data). Thirdly, a standardized serotyping method is needed as a gold standard to detect serotypes in Indonesia. Last, the non-vaccine type serotype (e.g., serotype 34) needs to be monitored.

## 5. Conclusions

The prevalence of pneumococcal NPC in Indonesia ranges from 21.0% to 87.6% among healthy children and from 9.2% to 73% among children with illnesses. The most frequently identified pneumococcal serotypes were 19F, 23F, 6B, 14, 19A, and 34. PCV13, currently used in the national program, covers 50.2% of dominant serotypes, while PCV20 offers 57.0%. Strategic subgroup analysis shows that coverage was lower in children with illnesses compared to healthy children. Given the limited data, active periodic surveillance is required to monitor post-vaccination serotype shifts, non-typeable strains, and vaccine escape, ensuring sustained effectiveness of Indonesia’s national immunization program.

## Figures and Tables

**Table 1:** Characteristics of the included studies.

No.	Author(s) (Year)	Study Period	Area	Population	Age	Specimen Type	Serotype/ Serogroup	*N*	NPC
1	Soewignjo et al. [20] (2001)	1997	Lombok	HC	0–25 months	NP	6, 23, 15, 33, 12, 19, 3, 14, 9, 18, 4, 10, 7, 11, 17, 22, NT	484	48%
2	Yuliarti et al. [25] (2012) *	2008– 2009	Jakarta	IC	28 days–60 months	B	7F	205	–
3	Farida et al. [26] (2014) ^†^	2010	Semarang	HC	6–60 months and 45–70 years	NP	Children: 6A/6B, 15B/C, 11A, 23F, 19F, 23A, 15A, others, NT Adults: 6A/6B, 15B/C, 11A, 23F, 15A, others, NT	496 (243 children and 253 adults)	27% (children 45% and adults 11%)
4	Safari et al. [27] (2014)	2012	Jakarta	IC	4–144 months	NP	19F, 19A, 6A/B, 23F, 11A, 9V, sg18, 12F, 15B/C, 3, 35B, 35F, 7F, NT	90	46%
5	Hadinegoro et al. [28] (2016)	2012	Central Lombok	HC	1–72 months	NP	6A/B, 19F, 23F, 15B/C, 19A, 14, 11A, 10A, 35B, 18, 34, 22F, 35F, 15A, 3, 20, 31, 38, 4, 17F, 7F, 1, 12F, 33F, 7C, 9V, NT	1200	46%
6	Dunne et al. [29] (2018)	2018	Bandung, Padang, Central Lombok	HC	12–24 months	NP	15B/C, 23F, NT2, 19F, 6A, 34, 6B, 3, 14, 6C, 11A, 16F, 18C, 9V, 38, 17F, 19A, 19B, 23A, 35A	302	Bandung: 63%, Padang: 34%, and Central Lombok: 50%
7	Murad et al. [30] (2019)	2014– 2015	Bandung	HC	8–12 weeks	NP	6B, NT2, 19F, 23F, 34, 15B/C, 6C, 14, 11A, 3, 13, 18C, 23A, 15A, 16F, 19A, 20B, 35A, 10B, 35B, 6A, 4, 9N, NT3b, 22F, 33B, 9V, 1, 7F, 8, 45, 17F, 23B, 38, 39, 18A, 19B, 28F, 35F, 7C, 40, 10F, 12F, 15F, 17A, 28A, NT4b, NT2/NT3b	200	85.4%
8	Purwanto et al. [31] (2020)	2017	Jakarta	IC	2 months–77.5 years	CSF	6B	147 (40 children and 107 adult)	–
9	Prayitno et al. [32] (2021)	2018– 2019	Lombok	HC	2–18 months	NP	6A/6B, 15B/C, 23F, 19F, 14, 34, 23A, 13, 6C/6D, 11A/11D, 15A/15F, 16F, 3, 35F/47F, 10A, 19A, 42/35A/35C, 23B, 20, 35B, 24A/24F/24B, 17F, 21, 18A/C/F, 5, 22F/22A, 39, 38/25F/25A, 1, 37/33F/33A, 31, 40/7C/7B, NT	233	21%
10	Muktiarti et al. [33] (2021)	2013	Jakarta	IC	12–152 months	NP	23A, 34, 19F, 6F, 6B, 23F, 6A, 7C, 6C, 15C/15B/C, 9V/9A, 13, 39, 10A, 9N, 15A, 17F, 19B, 24A, 28A, 35A, NT	52	Pre-vaccination period: 52%
11	Salsabila et al. [34] (2022)	2019	Kotabaru	HC	0–59 months	NP	6A/6B, 15B/C, 19F, 34, 23F, 35A/35C/42, 14, 19A, 13, 16F, 11A/11D, 7C/7B/40, 3, 4, 18C/18F/18B, 39, 17F, 22F/22A, 23B, 33F/33A/37, 35B, 6C/6D, NT	399	45%
12	Wahyono et al. [35] (2021)	2018– 2019	Banyumas	IC	6–12 years	NP	23A, 6A/6B, 3, 14, 6C/6D, 11A/11D, 15B/15C, 35B, 4, 20, 19A, 19F, 23F, 38/25F/25A, 15A/15F, sg18, 22F/22A, 35A/35C/42, 13, 34, 35F/47F, 7C/7B/40, 9V/9A, NT	122	73%
13	Safari et al. [36] (2021) ^†^	2017	Tabanan	IC	<18 and ≥18 years	NP	6A/6B, 19F, 14, 23F, 17F, 15B/15C, sg18, 3, 34, 20, 35A/35C/42, 21, NT	200 (159 children and 41 adults)	18.5% ( children 22.6%, adults 2.4%)
14	Yani et al. [37] (2023)	2018– 2019	Padang	HC + IC	<5 years	NP	19F, 14, 34, 6C, 23F, 6A, 6B, 4, 11A/11D, 15B/15C, 18C/18F/18B/18A, 19A, 33F/33A/37, NT	130	Healthy children: 53%, children with illnesses: 9.2%
15	Safari et al. [24] (2024)	2017	Southwest Sumba, Gunungkidul	HC	<5 years	NP	6B, 19F, 23F, 6C, 19A, 14, 11A, 6A, 34, 3, 13, 15B, 15C, 16F, 35B, 18C, 10A, 7C, 15A, 23A, 35A, 21, 22F, 6D, 20, 4, 7F, 8, 17F, 35F, 39, 42, 10B, 10F, 19B, 23B, 33B, 9V, 9A, 18A, 18B, 35C, 1, 2, 9N, 11D, 12F, 18F, 25A, 27, 28F, 31, 36, 37, 38, NT	1822	Southwest Sumba: 87.6% and Gunungkidul: 30.9%
16	Purwanto et al. [38] (2024) *^,†^	2019– 2020	Manado	IC	0–80 years	NP	Children: 14, 19A, 23F, 35B, 18C, 1, 6B, 6C, 31, 9V, 15C, 16F, 23A, 19F, NT Adult: 18C, 3, 17F, 35F, NT	106 (79 children and 27 adults)	29% (children 32.9% and adults 18.5%)
17	Daningrat et al. [39] (2024)	2018– 2019	Wakatobi	HC	<5 years	NP	23F, 6B, 19F, 6A,15B/C, 34, 11A, 6C, 14, 13, 19A, 3, 16F, 4, 23A, 10A, 18C/18F/18B, 21, 23B, 7F, 9V/9A, 35B, 7C/7B/40, 9A, 20, 15A, 17F, 18A, 31, 35F, 37, 42, 7A, NT	499	61.9%
18	Paramaiswari et al. [40] (2025)	2019– 2021	Jakarta	IC	≥6 years	NP	6C, 6B, 23F, 14, 3, 7C, 19A, 25A, 34, 19F, 16F, 42, 35B, 23B, 13, 15C, 6A, 11A, NT	50	Pre-vaccination period: 46%
19	Rani et al. [41] (2025) *	2024	Surabaya	IC	17 years	B	15A	1	–

Abbreviations: B: blood; CSF: cerebrospinal fluid; HC: healthy children; IC: children with illnesses; NESp: nonencapsulated *S. pneumoniae*; NP: nasopharyngeal; NPC: nasopharyngeal carriage; NT: non-typeable. * Studies based on blood or cerebrospinal fluid isolates were retained in the systematic review but excluded from the primary pooled NPC serotype coverage analysis and summarized descriptively. ^†^ For studies including both children and adults, only child-specific data were used when available.

**Table 2:** Estimated PCV serotype coverage among nasopharyngeal carriage studies.

Population Category	Number of Study Groups	Isolates Included	PCV10% (95% CI)	PCV13% (95% CI)	PCV15% (95% CI)	PCV20% (95% CI)
Healthy children, NPC	10	2583	40.9 (39.0–42.8)	50.7 (48.8–52.6)	51.4 (49.5–53.3)	58.0 (56.1–59.9)
Children with illnesses, NPC	7	210	31.9 (26.0–38.5)	43.3 (36.8–50.1)	43.3 (36.8–50.1)	45.2 (38.7–52.0)
Overall NPC studies	17	2793	40.2 (38.4–42.1)	50.2 (48.3–52.0)	50.8 (49.0–52.7)	57.0 (55.2–58.9)

Notes: NPC: nasopharyngeal carriage; PCV: pneumococcal conjugate vaccine; CI: confidence interval. Estimates represent descriptive pooled proportions of pneumococcal isolates or serotype-positive detections covered by each PCV formulation. The primary analysis was restricted to nasopharyngeal carriage studies. Blood and cerebrospinal fluid-based invasive pneumococcal disease studies were excluded from the primary pooled NPC analysis and summarized descriptively. Confidence intervals were calculated using the Wilson score method. Some studies contributed to more than one subgroup (e.g., healthy and illness populations) and were therefore analyzed as separate study groups, resulting in a total of 17 study groups.

**Table 3:** Multicolonization findings across included studies, including detection methods and study characteristics.

Study	Location	Method	Multicolonization (n)	Details
Soewignjo et al. [20]	Lombok	Culture	11	Not specified
Hadinegoro et al. [28]	Lombok	Culture	3	Not specified
Safari et al. [27]	Jakarta	PCR	1	Serotypes 3 and 9V
Murad et al. [30]	Bandung	PCR	93 (two serotypes), 5 (three serotypes)	–
Salsabila et al. [34]	Kotabaru	PCR	5	19F + 15B/15C; 6A/6B + NT; 19F + 17F; 16F + 6A/6B; 3 + 6A/6B
Wahyono et al. [35]	Banyumas	PCR	2	3 + 23A; 23F + 6C/6D
Yani et al. [37]	Padang	PCR	1	14 + 19A
Safari et al. [24]	Southwest Sumba & Gunungkidul	PCR	80 (two serotypes), 1 (three serotypes)	–
Daningrat et al. [39]	Wakatobi	PCR	39	Not specified

Notes: Multicolonization was defined as the detection of ≥2 pneumococcal serotypes within a single sample. Method refers to the serotype detection technique (PCR or culture) reported in each study. Some studies did not report detailed serotype combinations or exact counts.

**Table 4:** Risk of bias assessment using the JBI’s checklist for prevalence studies.

No.	Author(s) (Year)	Population Representativeness	Sampling and Data Collection	Measurement Reliability	Overall Risk of Bias
1	Soewignjo et al. (2001) [20]	Yes	Yes	Yes	Low
2	Yuliarti et al. (2012) [25]	Yes	Yes	Unclear	Moderate
3	Farida et al. (2014) [26]	Yes	Yes	Yes	Low
4	Safari et al. (2014) [27]	Yes	Unclear	Yes	Moderate
5	Hadinegoro et al. (2016) [28]	Yes	Yes	Yes	Low
6	Dunne et al. (2018) [29]	Yes	Yes	Yes	Low
7	Murad et al. (2019) [30]	Yes	Yes	Yes	Low
8	Purwanto et al. (2020) [31]	Unclear	Yes	Yes	Moderate
9	Prayitno et al. (2021) [32]	Yes	Yes	Yes	Low
10	Muktiarti et al. (2021) [33]	Unclear	Yes	Yes	Moderate
11	Salsabila et al. (2022) [34]	Yes	Yes	Yes	Low
12	Wahyono et al. (2021) [35]	Yes	Yes	Yes	Low
13	Safari et al. (2021) [36]	Unclear	Unclear	Yes	Moderate
14	Yani et al. (2023) [37]	Yes	Yes	Yes	Low
15	Safari et al. (2024) [24]	Yes	Yes	Yes	Low
16	Purwanto et al. (2024) [38]	Unclear	Yes	Yes	Moderate
17	Daningrat et al. (2024) [39]	Yes	Yes	Yes	Low
18	Paramaiswari et al. (2025) [40]	Unclear	Unclear	Yes	Moderate
19	Rani et al. (2025) [41]	Unclear	Unclear	Unclear	High

Notes: This table summarizes the key domains of the JBI checklist; detailed item-level assessments (Q1–Q9) are provided in Table S1.

## Data Availability

All data underlying this review are available in the published articles cited within the manuscript.

## Supplementary Materials

The following supporting information can be downloaded at: https://www.mdpi.com/article/10.3390/vaccines14050451/s1, Table S1: Detailed risk of bias assessment using the JBI’s checklist for prevalence studies.

## Abbreviations

The following abbreviations are used in this manuscript:

ARTI	Acute respiratory tract infection
ASEAN	Association of Southeast Asian Nations
B	Blood
CIs	Confidence interval
CSF	Cerebrospinal fluid
GRADE	Grading of Recommendations Assessment, Development and Evaluation
HC	Healthy children
HIV	Human immunodeficiency virus
IC	Children with illnesses
IPD	Invasive pneumococcal disease
JBI	Joanna Briggs Institute
Non-IPD	Noninvasive pneumococcal disease
Non-VT	Non-vaccine-type
NPC	Nasopharyngeal carriage
NT	Non-typeable
PCV	Pneumococcal conjugate vaccine
PPSV	Pneumococcal polysaccharide vaccine
PRISMA	Preferred reporting items for systematic reviews and meta-analyses
VT	Vaccine-type
