# Supplementary material for: Distribution of Streptococcus pneumoniae Serotypes in Nasopharyngeal Carriage Among Children in Indonesia and Estimated Coverage of Pneumococcal Conjugate Vaccines: A Systematic Review
Source: Vaccines (Basel). 2026 May 19;14(5):451. doi: 10.3390/vaccines14050451 (PMC13211392; doi:10.3390/vaccines14050451)
Supplement: Supplementary file 1 [file vaccines-14-00451-s001.zip › vaccines-4272353-supplementary.pdf]

Table S1. Detailed risk of bias assessment using the JBI's checklist for prevalence studies.

No.	Author(s) (Year)	Q1	Q2	Q3	Q4	Q5	Q6	Q7	Q8	Q9	Total Yes	Overall Risk of Bias
1	Soewignjo et al. (2001) [20]	Y	Y	Y	Y	Y	Y	Y	Y	Y	9	Low
2	Yuliarti et al. (2012) [25]	Y	U	U	Y	Y	Y	U	Y	NA	5	Moderate
3	Farida et al. (2014) [26]	Y	Y	Y	Y	Y	Y	Y	Y	Y	9	Low
4	Safari et al. (2014) [27]	N	N	U	Y	Y	Y	Y	Y	Y	6	Moderate
5	Hadinegoro et al. (2016) [28]	Y	Y	Y	Y	Y	Y	Y	Y	Y	9	Low
6	Dunne et al. (2018) [29]	Y	Y	Y	Y	Y	Y	Y	Y	Y	9	Low
7	Murad et al. (2019) [30]	Y	Y	Y	Y	Y	Y	Y	Y	Y	9	Low
8	Purwanto et al. (2020) [31]	U	U	U	Y	Y	Y	Y	Y	Y	6	Moderate
9	Prayitno et al. (2021) [32]	Y	Y	Y	Y	Y	Y	Y	Y	Y	9	Low
10	Muktiarti et al. (2021) [33]	N	N	N	Y	Y	Y	Y	Y	Y	6	Moderate
11	Salsabila et al. (2022) [34]	Y	Y	Y	Y	Y	Y	Y	Y	Y	9	Low
12	Wahyono et al. (2021) [35]	Y	Y	Y	Y	Y	Y	Y	Y	Y	9	Low
13	Safari et al. (2021) [36]	U	N	U	Y	Y	Y	Y	Y	Y	6	Moderate
14	Yani et al. (2023) [37]	Y	Y	Y	Y	Y	Y	Y	Y	Y	9	Low
15	Safari et al. (2024) [24]	Y	Y	Y	Y	Y	Y	Y	Y	Y	9	Low
16	Purwanto et al. (2024) [38]	U	N	U	Y	Y	Y	Y	Y	Y	6	Moderate
17	Daningrat et al. (2024) [39]	Y	Y	Y	Y	Y	Y	Y	Y	Y	9	Low
18	Paramaiswari et al. (2025) [40]	U	U	U	Y	Y	Y	Y	Y	Y	6	Moderate
19	Rani et al. (2025) [41]	U	U	N	Y	NA	U	U	NA	NA	1	High

Notes: Q1–Q9 correspond to the Joanna Briggs Institute (JBI) critical appraisal checklist for prevalence studies. Each item was rated as Y (Yes), N (No), U (Unclear), or NA (Not Applicable). JBI items: Q1. Was the sample frame appropriate to address the target population? Q2. Were study participants sampled in an appropriate way? Q3. Was the sample size adequate? Q4. Were the study subjects and the setting described in detail? Q5. Was the data analysis conducted with sufficient coverage of the identified sample? Q6. Were valid methods used for the identification of the condition? Q7. Was the condition measured in a standard, reliable way for all participants? Q8. Was there an appropriate statistical analysis? Q9. Was the response rate adequate, and if not, was the low response rate managed appropriately? Studies were categorized as low (≥7 "Yes"), moderate (4–6), or high risk of bias (≤3).
